# Crystal structure of (*E*)-2-[4-(4-hy­droxy­phen­yl)butan-2-yl­idene]hydrazine-1-carbo­thio­amide

**DOI:** 10.1107/S2056989014026401

**Published:** 2015-01-01

**Authors:** Adriano Bof de Oliveira, Johannes Beck, Christian Landvogt, Bárbara Regina Santos Feitosa, Fillipe Vieira Rocha

**Affiliations:** a Departamento de Química, Universidade Federal de Sergipe, Av. Marechal Rondon s/n, 49100-000 São Cristóvão-SE, Brazil; b Institut für Anorganische Chemie, Universität Bonn, Gerhard-Domagk-Strasse 1, D-53121 Bonn, Germany; c Instituto de Química, Universidade Estadual Paulista, Rua Francisco Degni s/n, 14801-970 Araraquara-SP, Brazil

**Keywords:** crystal structure, thio­semicarbazone, raspberry ketone, hydrogen bonding, three-dimensional

## Abstract

The title compound, C_11_H_15_N_3_OS, is a thio­semicarbazone derivative of the raspberry ketone rheosmin [systematic name: 4-(4-hy­droxy­phen­yl)butane-2-one]. The mol­ecule deviates from planarity, with the bridging C—C—C=N torsion angle equal to −101.3 (2)°. The maximum deviation from the mean plane of the non-H atoms of the thio­semicarbazone fragment [C=N—N—C(= S)—N] is 0.085 (5) Å for the Schiff base N atom, and the dihedral angle between this mean plane and the aromatic ring is 50.31 (8)°. In the crystal, mol­ecules are linked by N—H⋯O, N—H⋯S and O—H⋯S hydrogen bonds, forming a three-dimensional structure, with the mol­ecules stacked along [011].

## Related literature   

For one of the first reports of thio­semicarbazone derivatives synthesis, see: Freund & Schander (1902 ▸). For a report concerning the synthesis of the raspberry ketone, see: Hoffmann & Degner (1981 ▸). For the biological properties of thio­semicarbazone compounds as well as for their importance in coordination chemistry, see: Lobana *et al.* (2009 ▸).
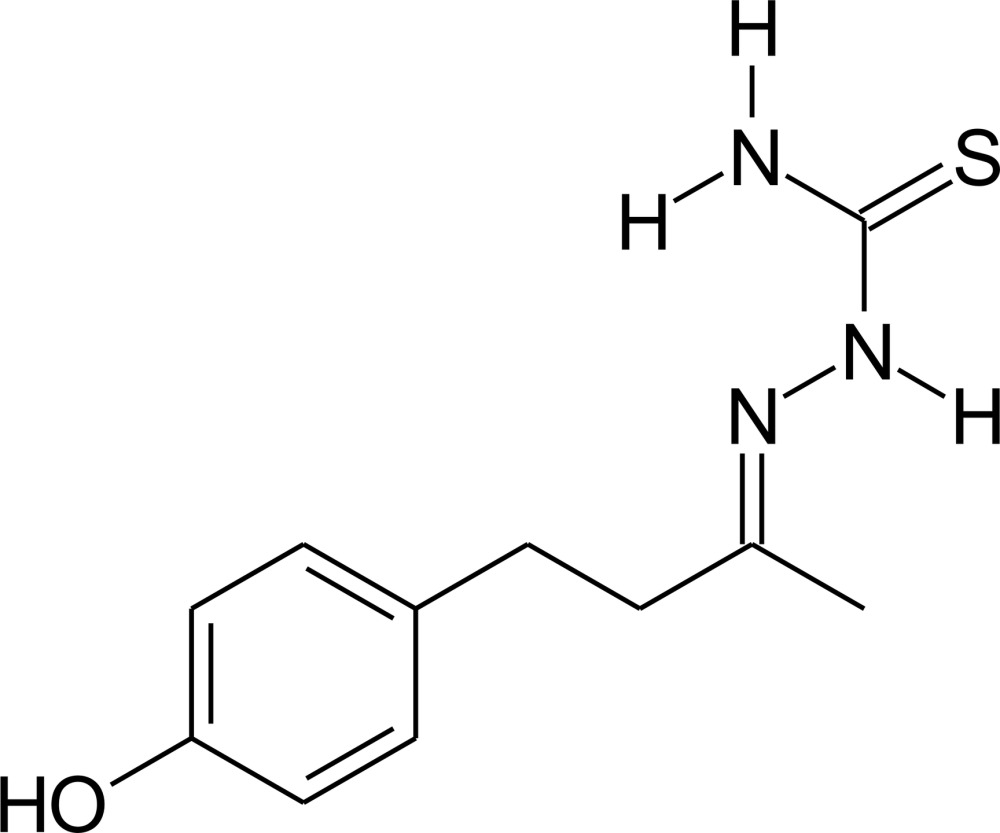


## Experimental   

### Crystal data  

C_11_H_15_N_3_OS
*M* *_r_* = 237.32
Monoclinic, 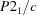

*a* = 13.5604 (7) Å
*b* = 9.7578 (6) Å
*c* = 9.3079 (4) Å
β = 95.194 (3)°
*V* = 1226.56 (11) Å^3^
*Z* = 4
Mo *K*α radiation
μ = 0.25 mm^−1^
*T* = 293 K
0.17 × 0.13 × 0.09 mm

### Data collection  

Nonius KappaCCD diffractometer
Absorption correction: multi-scan (Blessing, 1995 ▸) *T*_min_ = 0.929, *T*_max_ = 0.994
12737 measured reflections
2806 independent reflections
1587 reflections with *I* > 2σ(*I*)
*R*_int_ = 0.058

### Refinement  

*R*[*F* ^2^ > 2σ(*F* ^2^)] = 0.043
*wR*(*F* ^2^) = 0.116
*S* = 0.98
2806 reflections
205 parameters
All H-atom parameters refined
Δρ_max_ = 0.16 e Å^−3^
Δρ_min_ = −0.21 e Å^−3^

### 

Data collection: *COLLECT* (Nonius, 1998 ▸); cell refinement: *SCALEPACK* (Otwinowski & Minor, 1997 ▸); data reduction: *DENZO* (Otwinowski & Minor, 1997 ▸) and *SCALEPACK*; program(s) used to solve structure: *SUPERFLIP* (Palatinus & Chapuis, 2007 ▸); program(s) used to refine structure: *SHELXL97* (Sheldrick, 2008 ▸); molecular graphics: *DIAMOND* (Brandenburg, 2006 ▸); software used to prepare material for publication: *publCIF* (Westrip, 2010 ▸) and *WinGX* (Farrugia, 2012 ▸).

## Supplementary Material

Crystal structure: contains datablock(s) I, publication_text. DOI: 10.1107/S2056989014026401/su5031sup1.cif

Structure factors: contains datablock(s) I. DOI: 10.1107/S2056989014026401/su5031Isup2.hkl

Supporting information file. DOI: 10.1107/S2056989014026401/su5031Isup3.cml

**Fig. 1.**
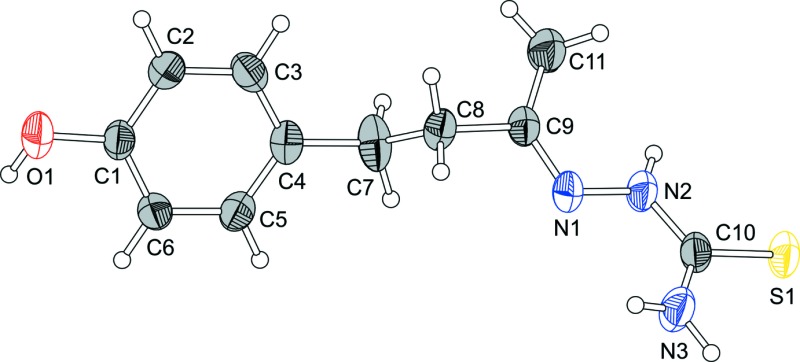
The molecular structure of the title compound, showing the atom labelling. Displacement ellipsoids are drawn at the 40% probability level.

. DOI: 10.1107/S2056989014026401/su5031fig1.tif
The mol­ecular structure of the title compound, showing the atom labelling. Displacement ellipsoids are drawn at the 40% probability level.

**Fig. 2.**
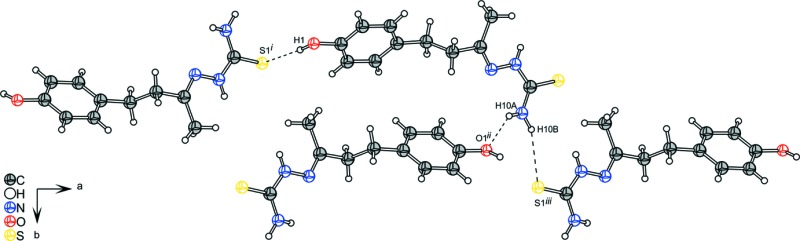
A view of the intermolecular hydrogen bonding (dashed lines) in the crystal of the title compound (see Table 1 for details of the hydrogen bonding and the symmetry codes).

**Fig. 3.**
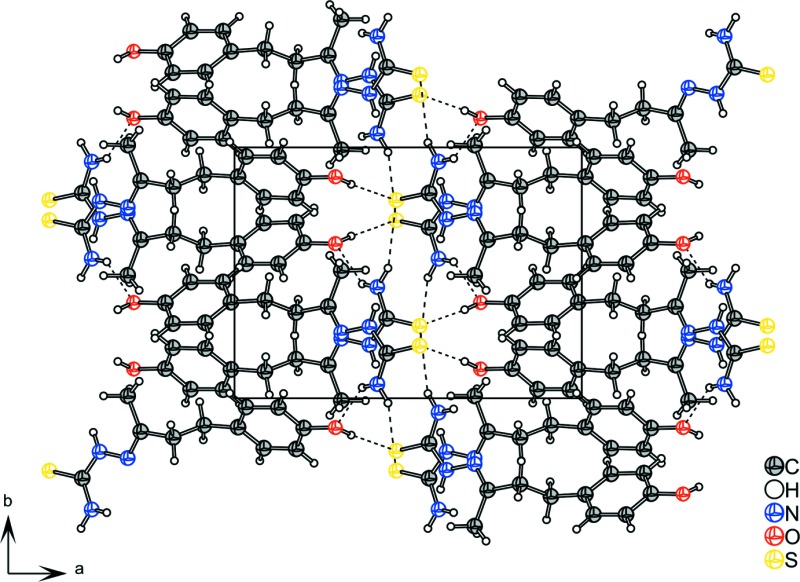
Crystal packing of the title compound viewed along the c axis, with the molecules stacking along the [011] direction. Hydrogen bonds are shown as dashed lines (see Table 1 for details).

. DOI: 10.1107/S2056989014026401/su5031fig2.tif
A view of the inter­molecular hydrogen bonding (dashed lines) in the crystal of the title compound (see Table 1 for details of the hydrogen bonding and the symmetry codes).

c . DOI: 10.1107/S2056989014026401/su5031fig3.tif
Crystal packing of the title compound viewed along the *c* axis, with the mol­ecules stacking along the [011] direction. Hydrogen bonds are shown as dashed lines (see Table 1 for details).

CCDC reference: 1036979

Additional supporting information:  crystallographic information; 3D view; checkCIF report

## Figures and Tables

**Table 1:** Hydrogen-bond geometry (, )

*D*H*A*	*D*H	H*A*	*D* *A*	*D*H*A*
O1H1S1^i^	0.89(4)	2.32(4)	3.206(2)	175(3)
N3H10*A*O1^ii^	0.85(2)	2.22(2)	2.936(2)	143(2)
N3H10*B*S1^iii^	0.91(3)	2.73(3)	3.585(2)	156.6(19)

Symmetry codes: (i) 
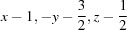
; (ii) 
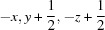
; (iii) 
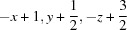
.

## supplementary crystallographic information

### S1. Structural commentary

Our work is actually dedicated to the synthesis and structural determination of thio­semicarbazone derivatives of natural products. The thio­semicarbazone unit is well known for its biological properties as well as for its importance in coordination chemistry (Lobana *et al.*, 2009). Herein, we contribute to the thio­semicarbazone chemistry by the synthesis and crystal structure of raspberry ketone thio­semicarbazone. The raspberry ketone is a natural product with great demand on the market and its synthesis has already been reported and optimized (Hoffmann & Degner, 1981).

In the title molecule, Fig. 1, the thio­semicarbazone unit is nearly planar showing a torsion angle for the N1—N2—C10—N3 entity of -3.1 (3)°. The maximum deviation from the mean plane of the non-H atoms of the C9/C10/N1/N2/N3/S1 fragment amounts to 0.085 (5)°. The angle between this mean plane and the aromatic ring is 50.31 (8)°. This strong tilting is possiblly due to free rotation around the *sp*^3^-hybridized C7 and C8 atoms (Fig. 1).

In the crystal, molecules are connected by N—H···O, N—H···S and O—H···S hydrogen bonds, with bridging sulfur atoms, into a three-dimensional H-bonded network (Figs. 2 and 3, and Table 1). The molecules are arranged along the [011] direction, but the hydrogen bonding inter­actions are present along all three directions (Fig. 3).

### S2. Synthesis and crystallization

The synthesis of the title compound was adapted from a procedure reported previously (Freund & Schander, 1902). In a hydro­chloric acid catalyzed reaction, a mixture of 4-(4-hy­droxy­phenyl)-2-butanone (raspberry ketone) (10 mmol) and thio­semicarbazide (10 mmol) in ethanol (80 mL) was refluxed for 5 h. After cooling and filtering, the title compound was obtained. Yellow crystals suitable for X-ray diffraction were obtained by slow evaporation of asolution in methanol.

### S3. Refinement

Crystal data, data collection and structure refinement details are summarized in Table 1. All the hydrogen atoms were located in a difference Fourier map and freely refined.

### Crystal data

**Table:**

C_11_H_15_N_3_OS	*F*(000) = 504
*M**_r_* = 237.32	*D*_x_ = 1.285 Mg m^−^^3^
Monoclinic, *P*2_1_/*c*	Mo *K*α radiation, λ = 0.71073 Å
Hall symbol: -P 2ybc	Cell parameters from 4899 reflections
*a* = 13.5604 (7) Å	θ = 2.9–27.5°
*b* = 9.7578 (6) Å	µ = 0.25 mm^−^^1^
*c* = 9.3079 (4) Å	*T* = 293 K
β = 95.194 (3)°	Rod, yellow
*V* = 1226.56 (11) Å^3^	0.17 × 0.13 × 0.09 mm
*Z* = 4	

### Data collection

**Table:**

Nonius KappaCCD diffractometer	2806 independent reflections
Radiation source: fine-focus sealed tube, Nonius KappaCCD	1587 reflections with *I* > 2σ(*I*)
Graphite monochromator	*R*_int_ = 0.058
Detector resolution: 9 pixels mm^-1^	θ_max_ = 27.5°, θ_min_ = 3.0°
CCD rotation images, thick slices scans	*h* = −15→17
Absorption correction: multi-scan (Blessing, 1995)	*k* = −11→12
*T*_min_ = 0.929, *T*_max_ = 0.994	*l* = −12→9
12737 measured reflections	

### Refinement

**Table:**

Refinement on *F*^2^	Primary atom site location: structure-invariant direct methods
Least-squares matrix: full	Secondary atom site location: difference Fourier map
*R*[*F*^2^ > 2σ(*F*^2^)] = 0.043	Hydrogen site location: difference Fourier map
*wR*(*F*^2^) = 0.116	All H-atom parameters refined
*S* = 0.98	*w* = 1/[σ^2^(*F*_o_^2^) + (0.0576*P*)^2^] where *P* = (*F*_o_^2^ + 2*F*_c_^2^)/3
2806 reflections	(Δ/σ)_max_ = 0.001
205 parameters	Δρ_max_ = 0.16 e Å^−^^3^
0 restraints	Δρ_min_ = −0.21 e Å^−^^3^

### Special details

**Table:**

Geometry. All e.s.d.'s (except the e.s.d. in the dihedral angle between two l.s. planes) are estimated using the full covariance matrix. The cell e.s.d.'s are taken into account individually in the estimation of e.s.d.'s in distances, angles and torsion angles; correlations between e.s.d.'s in cell parameters are only used when they are defined by crystal symmetry. An approximate (isotropic) treatment of cell e.s.d.'s is used for estimating e.s.d.'s involving l.s. planes.
Refinement. Refinement of *F*^2^ against ALL reflections. The weighted *R*-factor *wR* and goodness of fit *S* are based on *F*^2^, conventional *R*-factors *R* are based on *F*, with *F* set to zero for negative *F*^2^. The threshold expression of *F*^2^ > σ(*F*^2^) is used only for calculating *R*-factors(gt) *etc*. and is not relevant to the choice of reflections for refinement. *R*-factors based on *F*^2^ are statistically about twice as large as those based on *F*, and *R*- factors based on ALL data will be even larger.

### Fractional atomic coordinates and isotropic or equivalent isotropic displacement parameters (Å^2^)

**Table:**

	*x*	*y*	*z*	*U*_iso_*/*U*_eq_	
S1	0.53385 (4)	−0.71017 (6)	0.78232 (6)	0.0603 (2)	
O1	−0.29137 (12)	−0.88281 (19)	0.09365 (18)	0.0714 (5)	
N1	0.30776 (11)	−0.76195 (18)	0.49369 (17)	0.0513 (4)	
N2	0.38871 (12)	−0.7859 (2)	0.59241 (18)	0.0522 (5)	
N3	0.41152 (14)	−0.5570 (2)	0.6114 (2)	0.0635 (5)	
C1	−0.19991 (14)	−0.8803 (2)	0.1707 (2)	0.0480 (5)	
C2	−0.12722 (15)	−0.9634 (2)	0.1242 (2)	0.0538 (5)	
C3	−0.03396 (16)	−0.9638 (2)	0.1973 (2)	0.0550 (5)	
C4	−0.01049 (14)	−0.8834 (2)	0.3178 (2)	0.0493 (5)	
C5	−0.08500 (15)	−0.8014 (2)	0.3617 (2)	0.0515 (5)	
C6	−0.17858 (16)	−0.7981 (2)	0.2904 (2)	0.0496 (5)	
C7	0.08871 (17)	−0.8918 (4)	0.4046 (3)	0.0701 (7)	
C8	0.17714 (15)	−0.8434 (3)	0.3288 (2)	0.0501 (5)	
C9	0.27054 (13)	−0.8668 (2)	0.42605 (19)	0.0467 (5)	
C10	0.43922 (14)	−0.6808 (2)	0.6538 (2)	0.0493 (5)	
C11	0.3082 (2)	−1.0096 (3)	0.4435 (3)	0.0671 (7)	
H1	−0.337 (3)	−0.853 (4)	0.148 (4)	0.145 (15)*	
H2	−0.1415 (17)	−1.020 (2)	0.035 (2)	0.077 (7)*	
H3	0.0147 (16)	−1.022 (2)	0.1646 (19)	0.054 (6)*	
H5	−0.0730 (17)	−0.750 (2)	0.449 (2)	0.069 (6)*	
H6	−0.2290 (15)	−0.741 (2)	0.3238 (19)	0.051 (6)*	
H7A	0.101 (2)	−0.985 (3)	0.439 (3)	0.114 (11)*	
H7B	0.091 (2)	−0.833 (3)	0.499 (3)	0.103 (9)*	
H8A	0.1836 (15)	−0.894 (2)	0.235 (2)	0.060 (6)*	
H8B	0.1737 (14)	−0.751 (2)	0.3086 (19)	0.048 (6)*	
H9	0.4068 (16)	−0.864 (2)	0.614 (2)	0.054 (7)*	
H10A	0.3683 (18)	−0.544 (2)	0.541 (3)	0.077 (8)*	
H10B	0.4429 (17)	−0.481 (3)	0.650 (2)	0.078 (7)*	
H11A	0.377 (2)	−1.011 (3)	0.431 (3)	0.101 (9)*	
H11B	0.275 (2)	−1.069 (3)	0.380 (3)	0.121 (11)*	
H11C	0.302 (2)	−1.039 (3)	0.539 (3)	0.119 (11)*	

### Atomic displacement parameters (Å^2^)

**Table:**

	*U*^11^	*U*^22^	*U*^33^	*U*^12^	*U*^13^	*U*^23^
S1	0.0393 (3)	0.0773 (4)	0.0609 (3)	−0.0021 (3)	−0.0135 (2)	−0.0007 (3)
O1	0.0395 (9)	0.0927 (13)	0.0788 (10)	−0.0011 (8)	−0.0123 (8)	−0.0169 (9)
N1	0.0360 (9)	0.0618 (11)	0.0537 (9)	0.0004 (8)	−0.0091 (7)	−0.0022 (8)
N2	0.0378 (9)	0.0564 (13)	0.0593 (10)	0.0007 (9)	−0.0129 (7)	−0.0022 (9)
N3	0.0545 (12)	0.0599 (14)	0.0713 (13)	−0.0051 (10)	−0.0212 (9)	−0.0006 (10)
C1	0.0352 (10)	0.0496 (13)	0.0583 (11)	−0.0043 (10)	−0.0016 (8)	0.0011 (9)
C2	0.0484 (13)	0.0553 (14)	0.0575 (12)	−0.0053 (11)	0.0028 (10)	−0.0094 (10)
C3	0.0425 (12)	0.0553 (14)	0.0681 (14)	0.0078 (11)	0.0096 (10)	0.0020 (11)
C4	0.0388 (11)	0.0577 (14)	0.0504 (11)	−0.0057 (10)	−0.0006 (8)	0.0100 (9)
C5	0.0470 (12)	0.0546 (13)	0.0522 (12)	−0.0092 (11)	0.0007 (9)	−0.0043 (10)
C6	0.0403 (11)	0.0470 (13)	0.0618 (12)	0.0007 (10)	0.0052 (9)	−0.0023 (10)
C7	0.0400 (13)	0.107 (2)	0.0618 (14)	−0.0023 (13)	−0.0043 (10)	0.0199 (15)
C8	0.0413 (12)	0.0546 (14)	0.0525 (12)	−0.0065 (10)	−0.0067 (9)	0.0027 (10)
C9	0.0360 (11)	0.0555 (14)	0.0476 (11)	−0.0003 (10)	−0.0015 (8)	0.0010 (9)
C10	0.0328 (10)	0.0634 (15)	0.0513 (11)	−0.0021 (10)	0.0016 (8)	−0.0045 (10)
C11	0.0553 (16)	0.0638 (16)	0.0787 (18)	0.0031 (13)	−0.0125 (13)	−0.0031 (13)

### Geometric parameters (Å, º)

**Table:**

S1—C10	1.6973 (19)	C4—C5	1.379 (3)
O1—C1	1.376 (2)	C4—C7	1.507 (3)
O1—H1	0.89 (4)	C5—C6	1.378 (3)
N1—C9	1.281 (2)	C5—H5	0.95 (2)
N1—N2	1.386 (2)	C6—H6	0.95 (2)
N2—C10	1.333 (3)	C7—C8	1.520 (3)
N2—H9	0.82 (2)	C7—H7A	0.98 (3)
N3—C10	1.315 (3)	C7—H7B	1.05 (3)
N3—H10A	0.85 (2)	C8—C9	1.506 (2)
N3—H10B	0.91 (3)	C8—H8A	1.01 (2)
C1—C2	1.376 (3)	C8—H8B	0.92 (2)
C1—C6	1.382 (3)	C9—C11	1.488 (3)
C2—C3	1.381 (3)	C11—H11A	0.95 (3)
C2—H2	1.00 (2)	C11—H11B	0.91 (3)
C3—C4	1.383 (3)	C11—H11C	0.95 (3)
C3—H3	0.94 (2)		
			
C1—O1—H1	110 (2)	C1—C6—H6	119.7 (11)
C9—N1—N2	116.39 (18)	C4—C7—C8	115.97 (18)
C10—N2—N1	120.0 (2)	C4—C7—H7A	110.2 (17)
C10—N2—H9	118.6 (14)	C8—C7—H7A	109.0 (18)
N1—N2—H9	121.4 (14)	C4—C7—H7B	112.1 (15)
C10—N3—H10A	121.7 (16)	C8—C7—H7B	104.6 (15)
C10—N3—H10B	121.1 (15)	H7A—C7—H7B	104 (2)
H10A—N3—H10B	117 (2)	C9—C8—C7	109.24 (17)
O1—C1—C2	117.58 (18)	C9—C8—H8A	108.1 (12)
O1—C1—C6	122.95 (19)	C7—C8—H8A	112.5 (12)
C2—C1—C6	119.47 (18)	C9—C8—H8B	107.2 (12)
C1—C2—C3	119.8 (2)	C7—C8—H8B	111.8 (12)
C1—C2—H2	119.8 (13)	H8A—C8—H8B	107.9 (17)
C3—C2—H2	120.4 (13)	N1—C9—C11	125.35 (18)
C2—C3—C4	122.1 (2)	N1—C9—C8	116.50 (19)
C2—C3—H3	118.7 (11)	C11—C9—C8	118.01 (19)
C4—C3—H3	119.2 (11)	N3—C10—N2	117.12 (19)
C5—C4—C3	116.66 (18)	N3—C10—S1	122.95 (16)
C5—C4—C7	121.0 (2)	N2—C10—S1	119.93 (17)
C3—C4—C7	122.2 (2)	C9—C11—H11A	109.3 (18)
C6—C5—C4	122.55 (19)	C9—C11—H11B	112.3 (19)
C6—C5—H5	118.4 (14)	H11A—C11—H11B	110 (3)
C4—C5—H5	118.8 (14)	C9—C11—H11C	108.9 (19)
C5—C6—C1	119.4 (2)	H11A—C11—H11C	106 (2)
C5—C6—H6	120.8 (11)	H11B—C11—H11C	110 (3)
			
C9—N1—N2—C10	171.64 (19)	C2—C1—C6—C5	−0.4 (3)
O1—C1—C2—C3	179.56 (19)	C5—C4—C7—C8	−118.0 (3)
C6—C1—C2—C3	0.0 (3)	C3—C4—C7—C8	66.8 (3)
C1—C2—C3—C4	0.4 (3)	C4—C7—C8—C9	−176.4 (2)
C2—C3—C4—C5	−0.3 (3)	N2—N1—C9—C11	−1.1 (3)
C2—C3—C4—C7	175.1 (2)	N2—N1—C9—C8	174.63 (17)
C3—C4—C5—C6	−0.1 (3)	C7—C8—C9—N1	−101.3 (2)
C7—C4—C5—C6	−175.6 (2)	C7—C8—C9—C11	74.7 (3)
C4—C5—C6—C1	0.5 (3)	N1—N2—C10—N3	−3.1 (3)
O1—C1—C6—C5	−179.97 (19)	N1—N2—C10—S1	176.21 (14)

### Hydrogen-bond geometry (Å, º)

**Table:**

*D*—H···*A*	*D*—H	H···*A*	*D*···*A*	*D*—H···*A*
O1—H1···S1^i^	0.89 (4)	2.32 (4)	3.206 (2)	175 (3)
N3—H10*A*···O1^ii^	0.85 (2)	2.22 (2)	2.936 (2)	143 (2)
N3—H10*B*···S1^iii^	0.91 (3)	2.73 (3)	3.585 (2)	156.6 (19)

Symmetry codes: (i) *x*−1, −*y*−3/2, *z*−1/2; (ii) −*x*, *y*+1/2, −*z*+1/2; (iii) −*x*+1, *y*+1/2, −*z*+3/2.
