# Supplementary figures and images for: Crystal structure of (E)-2-[4-(4-hy­droxy­phen­yl)butan-2-yl­idene]hydrazine-1-carbo­thio­amide
Source: Acta Crystallogr E Crystallogr Commun. 2015 Jan 1;71(Pt 1):o33–4. doi: 10.1107/S2056989014026401 (PMC4331873; doi:10.1107/S2056989014026401)

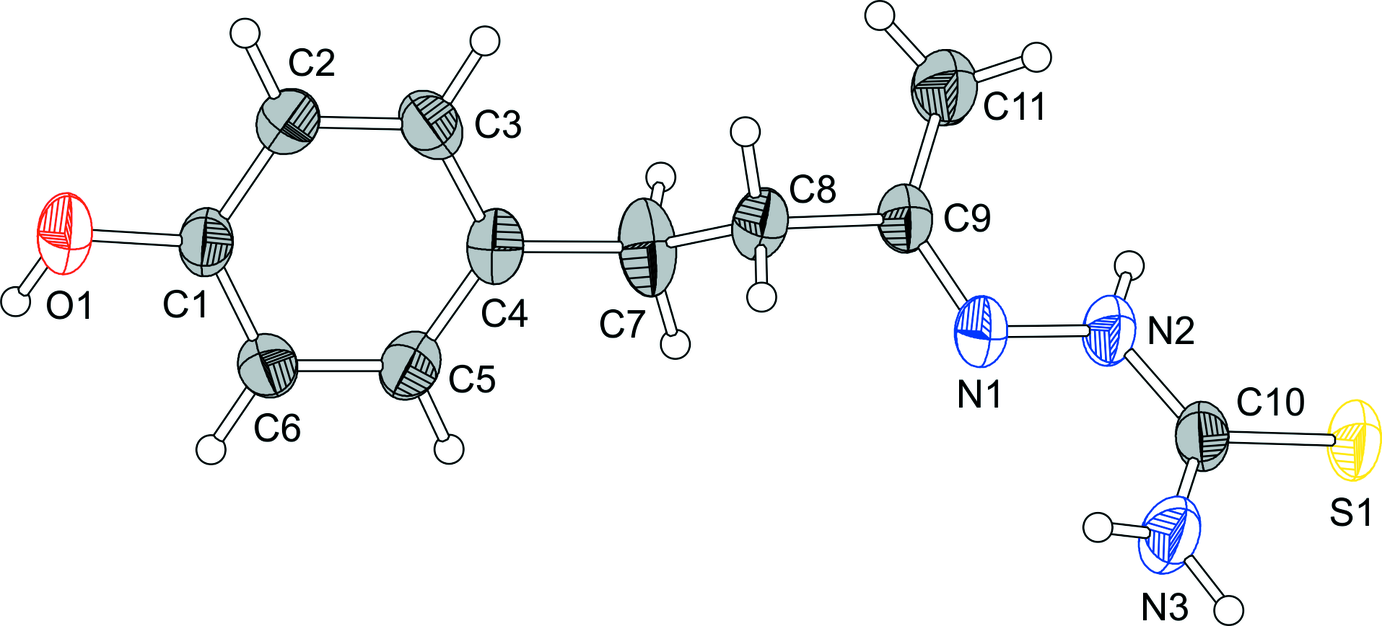

Supplement: Supplementary file 4 [file e-71-00o33-fig1.tif]

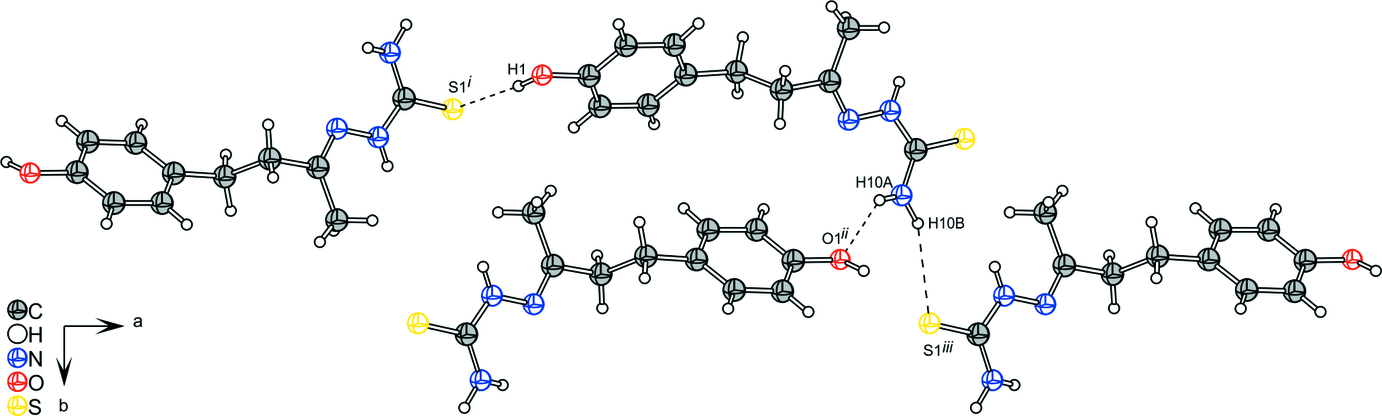

Supplement: Supplementary file 5 [file e-71-00o33-fig2.tif]

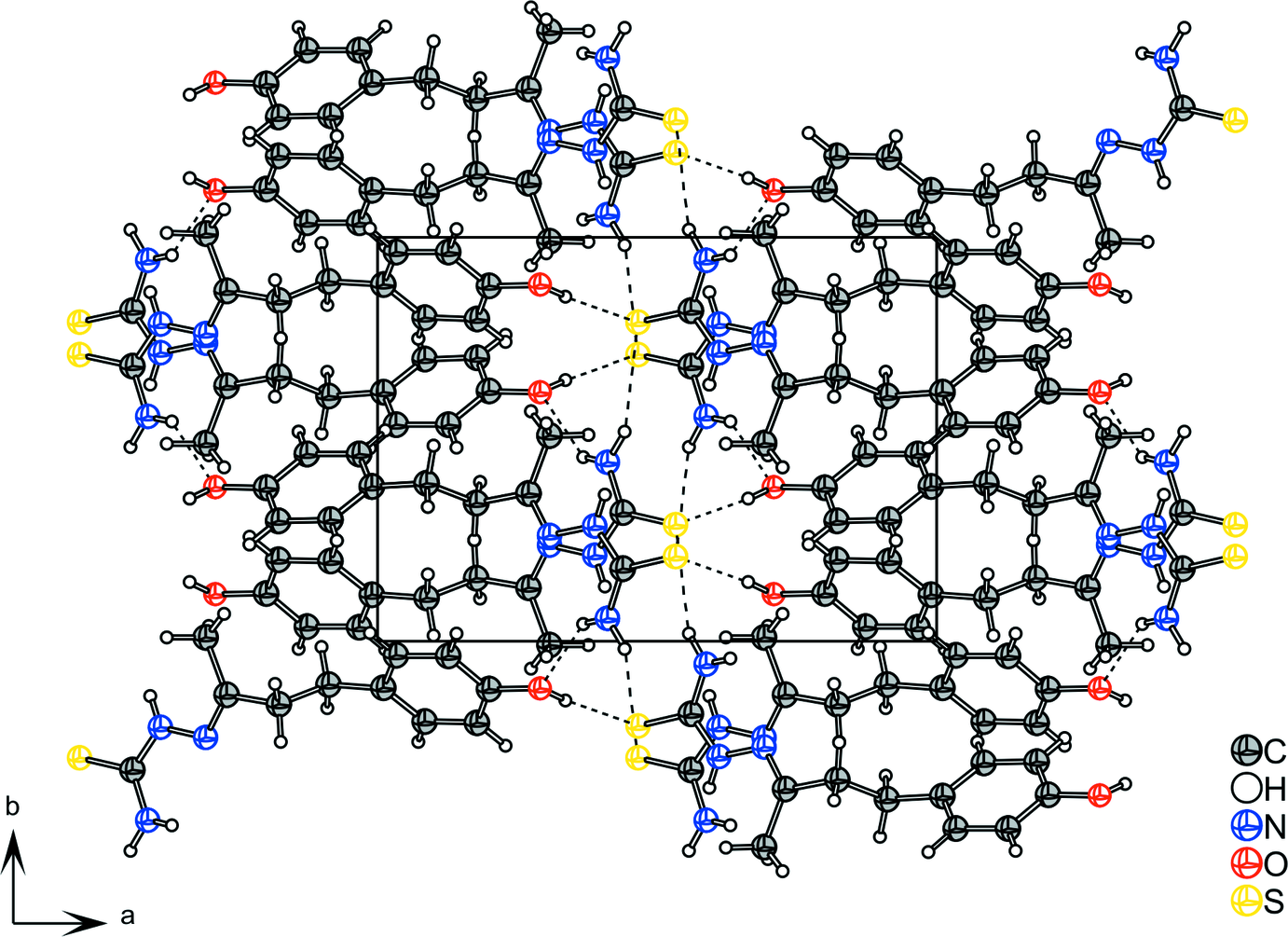

Supplement: Supplementary file 6 [file e-71-00o33-fig3.tif]
